# A comparative study on the grouping of CHS-DRG 2.0 and 1.1 versions based on empirical analysis

**DOI:** 10.3389/fpubh.2026.1853416

**Published:** 2026-08-11

**Authors:** Huiying Rong

**Affiliations:** The Second Hospital of Shanxi Medical University, Taiyuan, Shanxi, China

**Keywords:** China healthcare security diagnosis-related group, empirical analysis, grouping scheme, performance evaluation, version 1.1, version 2.0

## Abstract

**Objective:**

This study aims to compare and evaluate the grouping schemes and performance of China healthcare security diagnosis-related groups (CHS-DRG) versions 2.0 and 1.1, both based on DRG payment systems, in order to provide evidence for optimizing DRG grouping strategies and supporting the further advancement of DRG reform.

**Method:**

A total of 49,117 discharge records from a tertiary hospital in Shanxi Province, collected between January and July 2024, were analyzed. The coefficient of variation (CV) was employed to assess intra-group homogeneity, while the reduction in variation (RIV) was used to evaluate inter-group heterogeneity. Differences in grouping structure and performance between CHS-DRG version 2.0 and version 1.1 were systematically compared.

**Result:**

Following the transition from version 1.1 to version 2.0, the number of DRG groups increased by 7.13%. The proportion of ambiguous cases decreased by 54.86%, and the enrollment rate rose by 1.91%. After trimming, the proportion of groups with CV values below 1 increased by 0.65%. Additionally, the overall CV decreased from 0.37 to 0.36, while the RIV increased from 0.79 to 0.85.

**Conclusion:**

Compared with version 1.1, CHS-DRG version 2.0 demonstrates improved grouping performance. The updated scheme aligns more closely with clinical practice and is supported by more comprehensive policy frameworks. However, further coordination between medical service pricing reforms and DRG implementation remains necessary.

## 1 Introduction

Diagnosis-related group (DRG)-based payment is widely acknowledged internationally as a scientifically robust and advanced method for medical insurance reimbursement. It also functions as a key metric for assessing hospital performance, contributing significantly to the control of excessive growth in healthcare expenditures and to the improvement of hospital management efficiency. The China healthcare security DRGs (CHS-DRGs) system represents the localization of DRG payment principles within the context of China’s medical insurance framework. Developed in accordance with the characteristics of China’s healthcare service delivery and grounded in real-world medical cost data, it serves as the standardized and authoritative grouping system for DRG-based payment nationwide. The national healthcare security administration (NHSA) successively released CHS-DRG versions 1.0 and 1.1 in June 2020 and May 2021, respectively. Subsequently, in July 2024, the NHSA issued the *Notice on the Release of Version 2.0 Grouping Schemes for DRG and diagnosis-intervention packet (DIP), and on Further Promoting Related Work* (hereinafter referred to as the “Notice”) (1). This policy requires all regions to complete the transition to version 2.0 by January 1, 2025, with the aim of further improving the standardization and consistency of DRG-based payment systems.

Based on DRG case data collected from a tertiary general hospital in Shanxi Province between January and July 2024, this study systematically examines the key differences between CHS-DRG versions 2.0 and 1.1. The analysis aims to generate robust empirical evidence for evaluating the effectiveness of DRG-based medical insurance payment systems and to provide a practical reference for further policy optimization and refinement.

This study analyzed 49,117 discharge cases from a tertiary general hospital in Shanxi Province in 2024. The dataset was derived from audited and quality-controlled medical insurance settlement records, supplemented by DRG grouping results for CHS-DRG versions 1.1 and 2.0 provided by the medical insurance authority. The analysis primarily focused on comparing the two versions in terms of grouping categories and overall grouping performance.

## 2 Methods

Data were collected and organized using Microsoft Excel 2013, while statistical analyses were conducted with SPSS version 25.0. Outliers in hospitalization costs were identified and trimmed using the interquartile range (IQR) method. Grouping performance was assessed through multiple indicators, including enrollment rate, coefficient of variation (CV), and reduction in variance (RIV). Statistical comparisons were performed using the chi-square test and the Mann–Whitney *U* test, with a significance threshold set at *p* < 0.05.

In the implementation of DRG systems, the evaluation of grouping performance constitutes a critical basis for determining the extent to which a grouping scheme aligns with local healthcare conditions. Such evaluation further enables continuous optimization and refinement of the grouping framework based on empirical findings. Cost data within DRG groups typically exhibit a positively skewed distribution, making it necessary to identify and remove outliers prior to performance assessment. The IQR method is widely applied for trimming extreme values in hospitalization costs. However, given that different studies may adopt varying parameter settings for outlier detection, the selection of an appropriate trimming approach is of particular importance. Existing literature primarily relies on the Interquartile Range (IQR) method and Winsorization for outlier treatment, although the determination thresholds vary across studies. For example, both cited studies applied the IQR method to identify outliers (2, 3), but the latter modified the parameter used for the lower bound. Considering that inpatient expenditure data generally exhibit a pattern of “low-value concentration and high-value dispersion,” characterized by a pronounced right-skewed and long-tailed distribution, this study adopts the outlier identification formula used in the latter study in order to avoid misclassifying low-value observations as outliers.

Commonly used statistical indicators for assessing grouping performance include the CV and the RIV (4). The CV is primarily used to evaluate intra-group variability; lower values indicate greater homogeneity in case complexity or resource utilization within groups, reflecting better grouping performance. In general, a CV value of less than 1 is considered indicative of low intra-group heterogeneity (5). The RIV reflects the proportion of total variance explained by inter-group differences and is mainly used to assess heterogeneity between groups. In this context, the total sum of squares (TSSQ) represents the overall squared deviation from the mean across all observations, while the total within-group sum of squares (TWGSSQ) denotes the cumulative squared deviation within each group (6). Typically, an RIV value of 50–70% or higher is regarded as acceptable, with higher values indicating stronger inter-group discrimination and superior grouping performance (7). When subdividing adjacent DRGs (ADRGs) into specific DRGs, the process should adhere to the principle of minimizing intra-group variation while maximizing inter-group differences to the greatest extent possible. The corresponding equations are as follows:

$$\begin{array}{l} \text{Upper trimming limit}=\mathrm{Q3}+1.5\times \mathrm{IQR},\\ \text{Lower trimming limit}=Q1-0.5\times \mathrm{IQR} \end{array}$$

CV=Standard Deviation/Mean

RIV=(TSSQ−TWGSSQ)/TSSQ

## 3 Results

### 3.1 Comparison of enrollment rates

Among the 49,117 discharged cases, the number of ADRG groups increased from 332 under CHS-DRG version 1.1 to 358 under version 2.0, representing a 7.83% increase. Similarly, the number of DRG groups rose from 617 to 661, corresponding to an increase of 7.13%. The number of ambiguous cases decreased markedly, from 1,881 to 849, reflecting a reduction of 54.86%. In addition, the enrollment rate improved from 96.02 to 97.93%, representing an increase of 1.91% (Table 1).

**Table 1:** Comparison of enrollment rates between DRG versions 1.1 and 2.0.

Indicator	DRG1.1	DRG2.0	Comparison (%)
ADRG groups	332	358	7.83
DRG groups	617	661	7.13
Ambiguous cases	1881	849	54.86
Enrollment rate (%)	96.02	97.93	1.91

### 3.2 Comparison of enrollment types

In terms of enrollment status, the number of enrolled cases increased from 47,464 under CHS-DRG version 1.1 to 48,099 under version 2.0. In contrast, the number of unenrolled cases (including ambiguous cases) declined from 1,956 to 1,018, resulting in a higher overall enrollment rate in version 2.0. When stratified by treatment type (Internal Medicine, Procedure, and Surgery), the proportion of Procedure cases increased by 14.57%, whereas the proportion of Surgery cases decreased by 14.78%. Consequently, under version 2.0, both the Procedure and Internal Medicine categories accounted for larger shares, while the Surgery category represented a smaller proportion. With respect to complication and comorbidity (CC) classification (Non-CC, CC, MCC, and Unspecified CC), notable shifts were observed. The proportion of Non-CC cases increased substantially from 21.04 to 48.35%. In contrast, the proportions of CC, MCC, and Unspecified CC cases decreased from 31.70 to 19.46%, 12.40 to 9.37%, and 34.86 to 22.82%, respectively. Overall, the Non-CC category expanded, whereas all other categories contracted. The chi-square test indicated that these differences were statistically significant (*p* < 0.05) (Table 2).

**Table 2:** Comparison of enrollment types between DRG versions 1.1 and 2.0 (*n*, %).

DRG classification type		DRG1.1	DRG2.0	X^2^ value	P value
Enrollment status	Enrolled cases	47,161 (96.02)	48,099 (97.93)	305.08	0.000
	Unenrolled cases	1956 (3.98)	1,018 (2.07)		
Classification by treatment type	Internal medicine	19,180 (40.67)	19,661 (40.88)	4743.18	0.000
	Procedure	3,864 (8.19)	10,946 (22.76)		
	Surgery	24,117 (51.14)	17,492 (36.36)		
Classification by CC	Non-CC group	9,922 (21.04)	23,256 (48.35)	8333.76	0.000
	CC group	14,951 (31.70)	9,358 (19.46)		
	MCC group	5,848 (12.40)	4,507 (9.37)		
	Unspecified CC group	16,440 (34.86)	10,978 (22.82)		

CC–complication and comorbidity; MCC–major complication and comorbidity.

### 3.3 Differences in grouping efficacy

Regarding grouping efficacy, intra-group variability was assessed using the CV before data trimming. Under CHS-DRG versions 1.1 and 2.0, the proportion of DRG groups with CV < 1 increased from 88.67 to 90.23%, while the proportion with CV > 1 decreased from 11.33 to 9.77%. The overall CV declined from 0.6262 to 0.6102. However, the chi-square test indicated that this difference was not statistically significant (*p* > 0.05).

The Interquartile Range (IQR) method was utilized to trim outliers in hospitalization costs. Under CHS-DRG 1.1, the removed maximum and minimum values accounted for 4.86 and 4.79%, respectively; under Version 2.0, the removed maximum and minimum values were 5.00 and 4.66%.

After trimming outliers in hospitalization costs using the IQR method, the proportion of DRG groups with CV < 1 further increased from 97.67 to 98.32%, while the proportion with CV > 1 decreased from 2.33 to 1.68%. The overall CV value decreased from 0.3707 to 0.3640. The chi-square test again indicated that this difference was not statistically significant (*p* > 0.05) (Table 3).

**Table 3:** Comparison of grouping efficacy between DRG versions 1.1 and 2.0.

Indicator		Version 1.1	Version 2.0	X^2^	*P*
Pre-trimming	(*n*, %)	49,117(100.00)	49,117(100.00)		
Trimming	Extreme value (*n*, %)	2,389(4.86)	2,455(5.00)		
Trimming	Extremely low value (*n*, %)	2,354(4.79)	2,291(4.66)		
Post-trimming	(*n*, %)	44,374(90.34)	44,371(90.34)		
Pre-trimming				0.812	0.367
	Groups with CV < 1 (*n*, %)	532 (88.67)	586 (90.23)		
	Groups with CV > 1 (*n*, %)	68 (11.33)	14 (9.77)		
Post-trimming				0.686	0.408
	Groups with CV < 1 (*n*, %)	591 (97.67)	644 (98.32)		
	Groups with CV > 1 (*n*, %)	64 (2.33)	11 (1.68)		
Pre-trimming	CV value	0.6262	0.6102		
Post-trimming	CV value	0.3707	0.3640		

As shown in Table 4, before data trimming, the overall RIV under CHS-DRG versions 1.1 and 2.0 increased from 0.6108 to 0.6304. Major diagnostic categories (MDCs) exhibiting notable RIV increases included MDCS (Infectious & Parasitic Diseases), MDCN (Female Reproductive System Diseases), MDCF (Circulatory System Diseases), MDCE (Respiratory System Diseases), and MDCA (Pre-MDC). The Wilcoxon Signed-Rank Test indicated that the difference in RIV values between versions 2.0 and 1.1 before trimming was not statistically significant (*p* > 0.05).

**Table 4:** Comparison of RIV between DRG versions 1.1 and 2.0.

MDC code	Pre-trimming			Post-trimming		
	Version 1.1	Version 2.0	Difference	Version 1.1	Version 2.0	Difference
MDCA	0.6730	0.7066	0.0336	0.8105	0.8626	0.0521
MDCB	0.6516	0.6548	0.0032	0.8238	0.7931	−0.0307
MDCC	0.9853	0.9840	−0.0013	0.9918	0.9947	0.0029
MDCD	0.8296	0.8138	−0.0158	0.8918	0.9498	0.0580
MDCE	0.4838	0.5266	0.0428	0.7385	0.8548	0.1163
MDCF	0.6763	0.7221	0.0458	0.8288	0.9005	0.0717
MDCG	0.6241	0.6527	0.0286	0.7705	0.8576	0.0871
MDCH	0.4722	0.4968	0.0246	0.6028	0.7175	0.1147
MDCI	0.6208	0.6224	0.0016	0.7809	0.8434	0.0625
MDCJ	0.7400	0.7495	0.0095	0.8303	0.8838	0.0535
MDCK	0.4947	0.4981	0.0034	0.8301	0.8389	0.0088
MDCL	0.2581	0.2360	−0.0221	0.5557	0.7975	0.2418
MDCM	0.3298	0.3312	0.0014	0.6129	0.7825	0.1696
MDCN	0.5847	0.6639	0.0792	0.7140	0.8902	0.1762
MDCO	0.3022	0.2988	−0.0034	0.8526	0.9306	0.0780
MDCQ	0.1016	0.1010	−0.0006	0.2921	0.7017	0.4096
MDCR	0.4828	0.4910	0.0082	0.8028	0.8933	0.0905
MDCS	0.2012	0.3269	0.1257	0.2952	0.5843	0.2891
MDCT	0.9417	0.9424	0.0007	0.9743	0.9851	0.0108
MDCV	0.3722	0.3622	−0.0100	0.3783	0.4689	0.0906
MDCX	0.6242	0.6213	−0.0029	0.7195	0.7622	0.0427
MDCZ	0.5727	0.5708	−0.0019	0.7244	0.7434	0.0190
Z value	−1.899			−3.945		
*P-*value	0.058			0.000		
Total	0.6108	0.6346	0.0238	0.7911	0.8544	0.0633

MDCs with a small number of cases (MDCP, MDCU, MDCW, MDCY) were not included in the comparison.

After trimming outliers, the overall RIV increased from 0.7911 under version 1.1 to 0.8544 under version 2.0. MDCs with substantial increases included MDCQ (Blood & Immune Diseases), MDCS (Infectious & Parasitic Diseases), MDCL (Urinary System Diseases), MDCN (Female Reproductive System Diseases), and MDCM (Male Reproductive System Diseases). The Wilcoxon Signed-Rank Test showed that this post-trimming difference in RIV values was statistically significant (*p* < 0.05) (Table 4).

## 4 Discussion

### 4.1 Further optimization of DRG grouping

This study applied both CHS-DRG versions 2.0 and 1.1 to 49,117 cases, evaluating them in terms of enrollment rate, enrollment type, intra-group variability, and inter-group heterogeneity. Compared with version 1.1, the version 2.0 grouping scheme demonstrated clear optimization in its structure and application. The increased number and refinement of DRG groups allow for better alignment with the diagnosis and treatment of various diseases, facilitating more precise medical insurance payments and assisting healthcare institutions in controlling costs more effectively. In the present study, the number of ambiguous cases decreased by 54.86%, while the enrollment rate increased, enhancing both the scientific validity and accuracy of the grouping. Accurate enrollment also mitigates underpayment issues, ensuring reasonable compensation for medical services. Under version 2.0, the number of Procedure groups increased significantly, with a 14.57% rise observed, while Surgery groups decreased by 14.78%. Some DRG groups were reclassified from Surgery to Procedure, better reflecting non-operating room procedures. Additionally, the data showed an increase in the proportion of Non-CC cases and a decrease in the proportions of CC and MCC cases. This adjustment aligns with the control over CC/MCC cases implemented in version 2.0 and mirrors international trends in newer DRG versions. For example, in the 25th version of the US DRG system, the proportion of cases with comorbidities or complications decreased from 80 to 40% (8), enhancing the accuracy of case classification. Regarding performance indicators, both intra-group homogeneity and inter-group heterogeneity improved under version 2.0. The RIV values increased from 0.63 pre-trimming to 0.85 post-trimming, demonstrating stronger inter-group discrimination and enhanced grouping efficacy compared with version 1.1. These findings suggest that the version 2.0 scheme satisfies normative standards for rational inpatient case grouping in local hospitals and represents a significant improvement in grouping performance.

### 4.2 DRG grouping scheme more closely aligned with clinical practice

In addition to quantitative indicators, subjective evaluation plays an important role in assessing the effectiveness of DRG grouping, encompassing clinical rationality, feasibility, and administrative simplicity. The upgrade to CHS-DRG version 2.0, guided by pilot cities, healthcare institutions, and industry associations, incorporated expert input from multiple disciplines, including clinical medicine, statistics, coding, medical insurance, and hospital administration. By analyzing patterns in medical insurance data, version 2.0 achieved a more scientifically robust structure that aligns closely with actual clinical practice (9). The clinical validation process was significantly optimized. Independent validation by clinical specialty groups contributed to the establishment of a multidisciplinary joint validation model. The grouping results account for interrelated specialty patterns, improving consistency with real-world clinical scenarios. At the same time, the statistical methodology was upgraded from linear regression to a genetic algorithm model, and anesthesia risk stratification was introduced. These improvements better reflect clinical severity, reduce classification errors, and support the development of an optimized grouping scheme. Version 2.0 also introduced changes in group definitions and connotations, enabling the grouping scheme to more accurately represent actual diagnostic and treatment processes. For example, the new group EK1 (Invasive Ventilator Support < 96 Hours) captured cases that were previously classified under the ambiguous EQY group in version 1.1. Similarly, cases involving complex or combined procedures were reclassified; some cases originally in version 1.1 groups such as BC1 (Intracranial Vascular Procedures with Hemorrhage Diagnosis) and BE1 (Cervical and Cerebral Vascular Procedures) were reassigned to version 2.0 group BB1 (Complex Procedures of the Nervous System). This reclassification increased relative weights and payment standards, resulting in more reasonable and equitable medical insurance reimbursement.

### 4.3 More comprehensive supporting policies for DRG

Although the grouping efficacy of CHS-DRG version 2.0 has improved, this scheme alone cannot address all challenges in medical insurance payment reform. Accordingly, the Notice proposed complementary mechanisms, including special case review, negotiation and discussion, exclusion of certain items, clarification of settlement timelines, prepayments, and the establishment of medical insurance data working groups (10). In practice, regional authorities need to implement payment policies tailored to local conditions to support the DRG scheme. Such policies help standardize clinical diagnosis and treatment, accommodate technological advances in healthcare, safeguard the medical rights of insured individuals, alleviate financial pressure on healthcare institutions, enhance operational transparency, and strengthen the alignment between medical insurance and broader healthcare reforms. As payment management under medical insurance becomes increasingly precise, its capacity to optimize the structure and delivery of medical services is further enhanced.

### 4.4 Need for synergy between medical service price reform and DRG reform

In China, DRG-based payment in different regions relies on historical medical cost data rather than detailed cost accounting. This historical data is derived from prices of medical services, drugs, and consumables. Under the current conditions of zero markup for drugs and consumables, volume-based procurement, and price reductions for examinations and tests, it is particularly important to assess whether medical service prices adequately reflect the labor value of healthcare personnel. According to the requirements of the Pilot Plan for Deepening the Reform of Medical Service Pricing issued in 2021 by the NHSA and seven other departments (11), a medical service pricing mechanism that accurately reflects the technical labor value of healthcare personnel needs to be established. To promote standardization of national medical service price items, the NHSA released the *Guidelines for Initiating Medical Service Price Items* covering multiple disciplines. These guidelines, referencing items in the *National Technical Specification for Medical Service Items (2023 Edition)*, map and integrate existing price items to provide a reference framework for provincial implementation. The formulation and release of these guidelines provide a foundation for deepening the reform of medical service pricing. They restructure medical service price items, clarify policy directions, guide reasonable price adjustments, support the advancement of medical technologies, and emphasize the reflection of technical labor value and effort (12). From a long-term perspective, the interaction between payment reform, pharmaceutical pricing reform, and public hospital compensation reform is necessary to achieve systematic, coordinated, and synergistic policy effects (13). Such integration fosters value-based healthcare and contributes to achieving the goals of high-quality healthcare development and universal health coverage (14).

Overall, CHS-DRG version 2.0 represents a significant optimization over version 1.1. The updated scheme aligns more closely with clinical practice, integrates comprehensive supporting policies, and provides more refined management tools alongside a more rational payment mechanism. As a result, it encourages healthcare institutions to strengthen internal management, enhance the quality of medical services, and achieve both cost control and operational efficiency. As the data were derived solely from a single comprehensive tertiary hospital in Shanxi Province, this study is subject to certain limitations; future studies will endeavor to incorporate multi-center validation. Looking forward, future DRG grouping schemes are expected to further adapt to the practical demands of clinical work, serving as a key instrument in medical insurance reform and promoting the high-quality, sustainable development of both medical insurance and healthcare systems.

## Data Availability

The data analyzed in this study is subject to the following licenses/restrictions: due to the sensitive nature of the data (e.g., patient information), access is restricted. Requests to access these datasets should be directed to rose126mail@126.com.
